# Zinc oxide nano-fertilizer differentially effect on morphological and physiological identity of redox-enzymes and biochemical attributes in wheat (*Triticum aestivum* L.)

**DOI:** 10.1038/s41598-024-63987-9

**Published:** 2024-06-07

**Authors:** Muneeba Anum Nazir, Murtaza Hasan, Ghazala Mustafa, Tuba Tariq, Muhammad Mahmood Ahmed, Rosa Golzari Dehno, Mansour Ghorbanpour

**Affiliations:** 1 Department of Biotechnology, Faculty of Chemical and Biological Sciences, The Islamia University of Bahawalpur, Bahawalpur, 63100 Pakistan; 2 Department of Biotinformatics, Faculty of Chemical and Biological Sciences, The Islamia University of Bahawalpur, Bahawalpur, 63100 Pakistan; 3 Depatment of Plant Sciences, Faculty of Biological Sciences, Quaid-i-Azam University, Islamabad, 45320 Pakistan; 4 State Agricultural Ministry Laboratory of Horticultural Crop Growth and Development, Ministry of Agriculture, Department of Horticulture, Zhejiang University, Hangzhou, 310058 China; 5 Department of Agriculture, Chalus Branch, Islamic Azad University, Chalus, Iran; 6 Department of Medicinal Plants, Faculty of Agriculture and Natural Resources, Arak University, Arak, 38156-8-8349 Iran; 7 Institute of Nanoscience and Nanotechnology, Arak University, Arak, 38156-8-8349 Iran

**Keywords:** Nano-fertilizers, Wheat, Nanoparticle uptake, Zinc oxide NPs, Green synthesis, Physiology, Plant sciences

## Abstract

The aim of current study was to prepared zinc oxide nanofertilzers by ecofriendly friendly, economically feasible, free of chemical contamination and safe for biological use. The study focused on crude extract of *Withania coagulans* as reducing agent for the green synthesis of ZnO nano-particles. Biosynthesized ZnO NPs were characterized by UV–Vis spectroscopy, XRD, FTIR and GC–MS analysis. However, zinc oxide as green Nano fertilizer was used to analyze responses induced by different doses of ZnO NPs [0, 25, 50,100, 200 mg/l and Zn acetate (100 mg/l)] in *Triticum aestivum* (wheat). The stimulatory and inhibitory effects of foliar application of ZnO NPs were studied on wheat (*Triticum aestivum*) with aspect of biomass accumulation, morphological attributes, biochemical parameters and anatomical modifications. Wheat plant showed significant (*p *< 0.01) enhancement of growth parameters upon exposure to ZnO NPs at specific concentrations. In addition, wheat plant showed significant increase in biochemical attributes, chlorophyll content, carotenoids, carbohydrate and protein contents. Antioxidant enzyme (POD, SOD, CAT) and total flavonoid content also confirmed nurturing impact on wheat plant. Increased stem, leaf and root anatomical parameters, all showed ZnO NPs mitigating capacity when applied to wheat. According to the current research, ZnO NPs application on wheat might be used to increase growth, yield, and Zn biofortification in wheat plants.

## Introduction

Wheat is most cultivated crop and second most important cash nutritious crop in the whole world due to high nitrogen contents. It’s quite important because of its nutritional contents in the whole world as well^1^. Because of its caloric and protein values, 696 million mg wheat were produced, to fulfill the requirement of one third population of the whole world. Unfortunately, wheat production has not increased in last 50 years due to environmental and anthropogenic factors such as water, drought, temperature, abiotic stresses^2–4^ and human development factor which are very major concern ^5^. By the year 2023, World’s increasing population has become the major concern for providing food, shelter and survival. The pace of growing population is creating a lot of issues like place to live, food supplies, water, clothes, education and health ^6^. Improvement for wheat variety has been a great focus for researcher and scientists because wheat is the major crop in ^7^. During 1950 to 2020, due to increased food demand, fertilizer use has increased more than 13 times. This increase has led to enhanced soil contamination, increased agricultural cost and a little shortage of chemical fertilizers in market ^8^. The concern has led to the synthesis of a controlled technique to develop fertilizers that are environment friendly and doesn’t affect future consumers^9,10^. They use green method for synthesis and processing in plants leading to soil integrity and maintaining ecosystem with high crop yield ^11^. In past decade, nanotechnology has been used in various fields and marked its importance in every field including agroindustry^12–14^. The old traditional methods for improving the growth and quantity of crops is not sufficient to meet the demands of current increasing population level ^15^. The focus on synthesis and improvement of nano-fertilizers is of utmost importance to scientists and it has progressed significantly^16–18^. With its uniqueness, nanotechnology is revolutionizing every field including agriculture with their efficiency and enhancing the plants to absorb nutrients ^19^. In recent years, nanomaterials have been employed in agriculture, such as in the form of Nano fertilizers and nano pesticides^20–23^. Their application as fertilizers has contributed to an increase of agricultural production globally ^24^. Micronutrients Nano fertilizers consist of trace micro nutrients needed by plants like Titanium, Zinc, Nickel, Manganese, Boron, Iron, coper etc., Oxides of these elements are used for Nano fertilizers synthesis and proven to be quite effective in plant production^25–27^. Although, they are required in high amount as compared to macronutrients, but their production and efficiency is good as compared to the macronutrient fertilizer ^28^. Studies highlight zinc oxide nanoparticles (ZnO NPs) as generally safe for biological species, showing promising effects on stimulating seed germination, plant growth and defense from diseases ^29^. Nevertheless, research involving these NPs and their impact on plants growth and metabolism at different stages have returned mixed results. NPs features, as well as the anatomy of the host plant, determine how NPs are absorbed, translated, and accumulated ^30^. This study's primary objective was to list the uses of ZnO nanoparticles for agricultural production as well as investigate the biological synthesis, uptake, translation, and biotransformation of ZnO nanoparticles in plants. The reason behind choosing ZnO NPs is because of their efficiency, role of Zinc in human bodies and the concept of green agriculture that seems quite achievable by modifying our conventional cultivating ways. Studies have shown that ZnO NPs cast positive as well as negative effects on plant growth and metabolism. The excess use of Zinc nano-agrochemicals can be potentially toxic to soil microbes and their associated functions, such as nitrogen (N) mineralization and decomposition of organic materials^31,32^. Furthermore, the concentration of zinc is very important, increase the concentration enhanced the toxicity level which ultimately reduced the crop productivity. Moreover, the accumulation of nanoparticles (NPs) in edible crops may reduce food quality, and can cause serious threats to human health^33,34^. Uptake, accumulation and translocation of ZnO NPs by plants also vary according to the features and anatomy of the host plant. ZnO NPs' green production as well as plant absorption, transport, and biotransformation have all been well studied in research. The main aim of this research was to look at the effects of ZnO NPs with different concentration especially on *Triticum aestivum* plant. Secondly, investigation of morphological and anatomical changes in of *Triticum aestivum* in response to application of various concentrations of ZnO NPs. In the end, exploring the mechanism of ZnO NPs and metallic element zinc acetate on physiological changes and yield of *Triticum aestivum*.

## Material and methods

### Plant materials

*Triticum aestivum* seeds were purchased from The Islamia University of Bahawalpur's Cholistan Institute of Desert Studies (CIDS) Bahawalpur, Pakistan. To assure surface sterility, seeds were submerged in a 10% sodium hypochlorite solution for 10 min. After that, they were washed three times with DI-water. Then these seeds were used as plant model to study effects of ZnO NPs.

### Preparation of plant extract

Dry Seeds of *Withania coagulans* were purchased from local market of Bahawalpur city, Pakistan. The plant materials were washed with running tap water and then finally washed with distilled water to remove all impurities. Later on, these seeds were dried in sunlight first and then in oven until all the moisture were lost. In third step, all seeds were grounded by mortar and pestle to coarse fine powder. In the fourth step, 10 g of seeds powder was added into 200 ml of distilled water. This was boiled on hot plate stirrer for 45 min. Then cooled and filtered and then plant extract filtrate was used as reducing agent in preparation of Zinc Nanoparticles and were kept at 4 °C for future use.

### Synthesis of ZnO NPs

ZnO nanoparticles have been synthesized by green synthesis procedure as 50 ml of distilled water having 0.2 g Zinc acetate and 3 mL of extract of *Withania coagulans* as previously reported^35,36^. The zinc precursors with reducing agent were keep on magnetic stirrer temperature control process and keep pH of solution 12 with help of 2 M NaOH solution. The resulting mixture was vigorously stirred on hotplate for 5 h at 90 °C and observing the change of color. Uv–vis spectrophotometric analysis confirms synthesis of nanoparticles at 350 nm. After centrifugation of mixture at 6000 rpm for 10 min supernatant was discarded and pellets were dried in oven at 100 °C the white product was obtained.

### Characterization of ZnO NPs

The synthesized ZnO NPs were characterized by ultraviolet (UV)–visible spectroscopy, X-ray diffraction (XRD) and FTIR techniques.

#### Ultra violet–vis spectrometry (UV)

Ultra violet–visible (UV–Vis) spectrometry is a widely used analytical technique to determine the absorbance or transmission of light in the ultraviolet and visible regions of the electromagnetic spectrum. Epoch-BioTek Instruments USA is a well-known manufacturer of laboratory instruments, including UV–Vis spectrometers. This UV–Vis was used to *analyses role of Withania coagulans*, plant extracts as reducing agent and to confirm the synthesis of Zinc oxide nanoparticles.

#### X-ray diffraction analysis (XRD)

X-ray diffraction analysis (XRD) is a powerful technique used to determine the atomic and molecular structure of a crystalline material. Rigaku is a well-known manufacturer of X-Ray Diffractometer Systems, including the Ultima IV model that was used to analyses role of *W. coagulans*, based on ZnO NPs using Cu Kα radiation, all diffracted intensities were recorded at 40 kv and 30 mA current at range from 20 to 80 °C. All the green synthesized prepared NPs were tested with the help of X-ray diffraction analysis (XRD).

#### Scanning electron microscope (SEM)

Scanning electron microscope (SEM) combined with Energy Dispersive X-ray Spectroscopy (EDS) is a powerful micro-analysis system that allows for high-resolution imaging and elemental analysis of a wide range of Biologically prepared ZnO NPs samples. Therefore, the size and morphology of the ZnO, nanoparticles were analyzed using SEM analysis.

### Pot experiment

The experiment to study the response of wheat (*Triticum aestivum*) to different concentrations of zinc oxide nanoparticles was conducted under natural environmental conditions (28/20 °C day/night temperature, relative humidity 67 ± 5%) at the research area of the Cholistan Institute of Desert Studies (CIDS), The Islamia University of Bahawalpur, Pakistan during the Rabi season of 2021–2022. The recommended seeds variety was purchased from the local market and there were 10 seeds sowed in each pot, which were filled with 5.0 kg of soil, using a completely randomized design in triplicates. After germination, thinning was made and five plants were left in each pot.

Different amounts of NPs were chosen (25, 50, 100, 200 PPM) as treatments, and the ZnO NPs applied to the plants at various time intervals. Instead of this, all other condition such as humidity, temperature, moisture contents were setted as previous reports and modified ^37–39^. The green synthesized NPs solution was prepared using distilled water, and homogenized for around 30 min before application using ultrasonication. After, 30 days seeding, the first foliar spray of ZnO NPs was made. The second, third, and fourth sprays of NPs were carried out in the seventh, tenth, and eleventh weeks of sowing. Each pot was covered before each spray to prevent NPs contamination of the soil. Control plants without NPs inoculation were sprayed with distilled water. Each treatment utilized 100 mL of total NPs solution. Each treatment has three replicas. Over the course of 120 days, the soil moisture level was maintained at around 70% of its water holding capacity. Samples for morphological, anatomical and biometabolics analysis were gathered on a regular basis for various analyses. The treatment codes and their description for pot experiment are represented in Table 1.

**Table 1:** Treatment codes and their description for pot experiment.

S. no.	Treatment code	Treatment
1	T0	Growth in distilled water
2	T1	Growth in 25 mg/l of Zn oxide nano-particles
3	T2	Growth in 50 mg/l of Zn oxide nano-particles
4	T3	Growth in 100 mg/l of Zn oxide nano-particles
5	T4	Growth in 200 mg/l of Zn oxide nano-particles
6	T5	Growth in 100 mg/l of Zn Acetate Solution

### Plants harvesting

Plants *Triticum aestivum* under all treatment’s conditions with green synthesized zinc oxide NPs were collected after 120 days. After measuring weight, height, and spike length, the plants were divided into spikes, grains, shoots, and roots. The plant with roots, shoot and leaves were cleaned with distilled water after being treated with dilute HCl (0.1%) acid (to remove metals from the roots' surface). The weight of oven dried roots and shoots was calculated, and such materials were sliced into small bits for further investigation.

### Morphological observation of *Triticum aestivum* incubated ZnNPs

Morphological analysis was carried out at the end of experimental conditions, *Triticum aestivum* plants were harvested and washed by using MilliQ-water. *Triticum aestivum* growth and morphological changes were recorded with plant height in cm, shoot length cm, root length cm, leaf area cm^2^, number of leaves per plant, fresh weight of plant g, dry weight of plant g and leaf water content. All the plant samples were without delay frozen for further analysis at −80 °C.

### Photosynthetic pigments

Harvested plant in all treatment of different concentration and replicates were tested for the growth such chlorophyll contents. The content of photosynthetic pigments was determined using the method described by ^40^. Chlorophyll and carotenoids were recovered from a 1:5 diluted methanolic extract of leaves. Using a UV–vis spectrophotometer, absorbance was measured at 662 nm, 645 nm, and 652 nm for chlorophyll and 470 nm for carotenoids.

### Yield of *Triticum aestivum*

The grains estimation was carried out for the determination plant yield for the treatment of nanoparticles in different conditions. The parameters were recorded using standard protocols such as plant height (cm), number of leaves (Frequency), number of tillers, leaf areas (cm^2^), root length (cm), shoot length (cm), fresh weight(g), dry weight(g), spike length(cm), number of spikelets, number of grains per spike and 100 grain weight(g).

### Estimation of protein, proline

By grinding 1 g of seedling in 2 ml of phosphate buffer (pH 7.00), protein content was evaluated using the technique of ^41^. The extract was filtered, and 40 µL of it was placed in a separate test tube before being mixed with 160 µL of Bradford reagent. The solution was mixed with a vortex mixer, and the absorbance at 595 nm was determined. The proline content was calculated using the technique described by ^42^. In a cold pestle and mortar, 0.3 g of leaf tissue from control and treatment plants was homogenized by adding 5 ml of a 3% sulfosalicylic acid solution. Centrifuging the homogenates at 4000×*g* for 10–15 min at 4 °C. 2 ml of acid ninhydrin, 2 ml of 96% acetic acid, and 1 ml of 3% sulfosalicylic acid were mixed together in glass tubes for each sample. In these tubes, 0.3 g of the 2 ml supernatant from each homogenate was mixed to create acid ninhydrin. These tubes were incubated in a hot water bath for 1 h at 96 degrees. 4 ml of toluene were added to each tube after incubation. The absorbance of the upper phase of pink red was measured. The proline content of each sample was calculated using a proline standard curve.

### Anatomical analysis of *Triticum aestivum*

The sampling of *Triticum aestivum* was done of random plants in each plot. Pieces of leaves, stems and roots were taken with the help of scissor of about 2 cm. Three replicates of each plant were taken from each plot and control as well. All the plants were then rinsed under running water to eliminate any dust particles. FAE solution (5 ml formaldehyde, 5 ml acetic acid and 90 ml of 90% ethanol) was used for fixing these samples. The slides prepared were fixed and focused under light compound microscope at different resolution powers; viz 10× and 40× using digital camera of 20 mega pixels. Photographs were taken from microscope in laboratory Department of Botany, The Islamia University of Bahawalpur. Root, stem ad leaf characters of dermal, ground and vascular tissues were studied and measured in thickness at 10× in cm while cell area measured at 40× in cm^2^.

### Antioxidant enzyme activities of *Triticum aestivum*

POD, SOD and CAT was determined using the approach described in ^43^. 1 g of fresh leaf sample was homogenized in cold phosphate buffer with pH 7, centrifuged at 15,000 rpm for 15 min at 40 °C, and the supernatant was collected.

To test POX activity, a reaction mixture of 800 µl of phosphate buffer (pH 5.0), 100 µl of H_2_O_2_, and 100 µl of guaicol (20 mM) was produced. The reaction mixture was then combined with 100 µl of enzyme extract. For POD, the ultimate absorbance was measured for 2 min at 470 nm. The change in absorbance/min/mg protein was used to determine activity. To evaluate SOD activity, a total of 100 µl of enzyme extract was placed in a separate test tube, followed by 500 µl of SOD buffer, 200 µl of methionine, 200 µl of Triton X, 100 µl of NBT, and 800 µl of dH_2_O. The liquid was mixed with a vortex mixer before being exposed to a UV lamp for 15 min at 25 °C before adding 100 µl of riboflavin. The absorbance was then measured at 560 nm with a spectrophotometer.CAT activity was determined by combining enzyme extract with 5.9 mM H_2_O_2_ in a 1:1 ratio. The rate of disappearance of H_2_O_2_ at 240 nm is used to calculate CAT activity. For 2 min, absorbance decreased, and one unit of CAT activity was defined as mM H_2_O_2_ decomposed/min/mg protein.

### Data analysis

Each treatment was repeated three times, and the findings were provided as mean standard deviation. The one-way ANOVA analysis was used to analyze the experimental data, and the Tukey’s HSD post Hoc test was used to make multiple comparisons of means. Each experimental value was compared to the appropriate control value.

### Statement on experimental research and field studies on plants

The cultivated plants sampled comply with relevant institutional, national, and international guidelines and domestic legislation of Pakistan.

## Results and discussion

**Figure 1:**
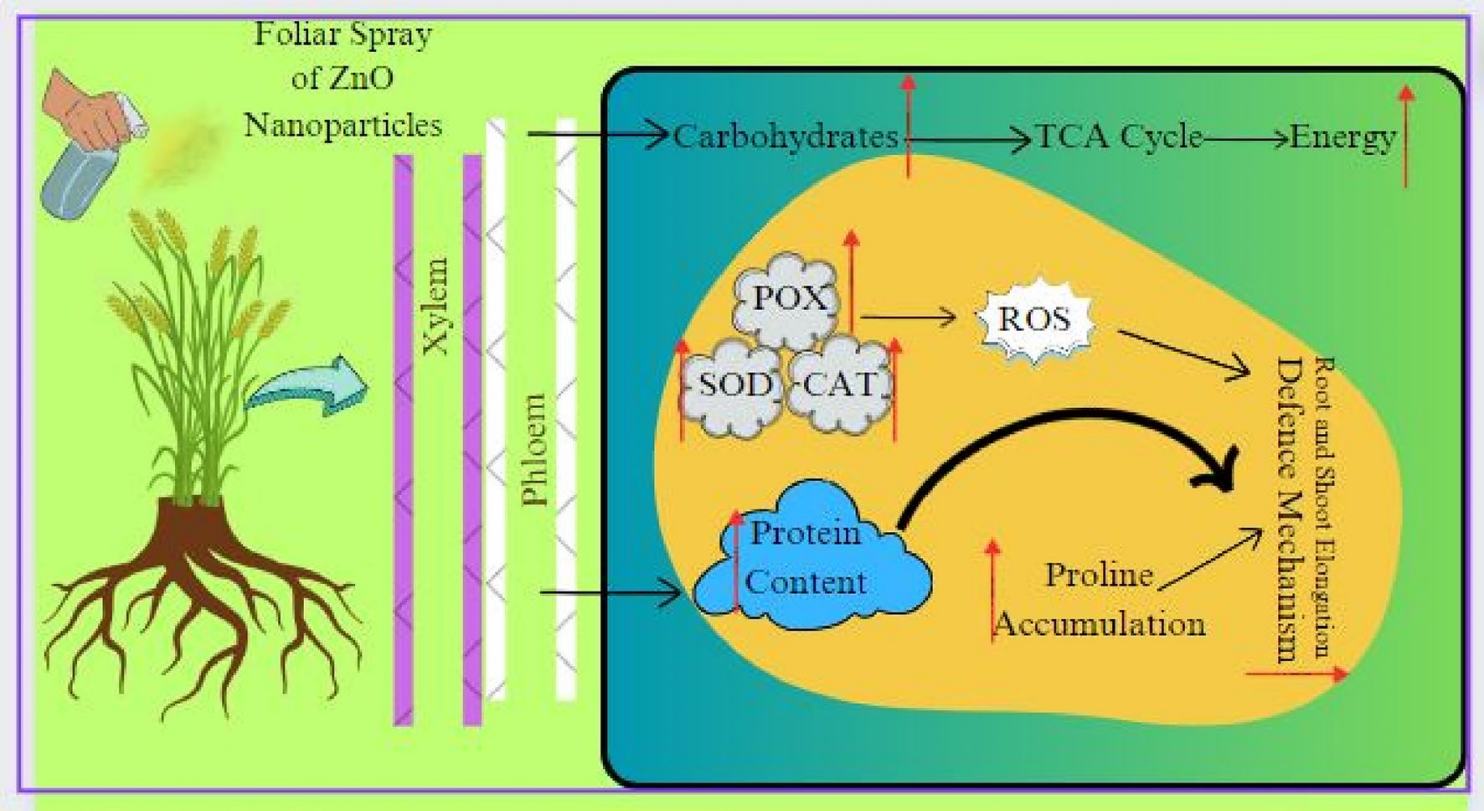
Schematic illustration of phyto-synthesized ZnO Nanoparticles and their performance on Wheat (morpho-anatomical and biochemical) properties.

Nanotechnology has a pronounced potential for achieving sustainable agriculture all over the world. Zn NPs performed better compared to conventional Zn fertilizers^44,45^. ZnO NPs significantly increased the physical parameters of wheat, rice, soya bean, barely and others cash crops compared to control under all kinds of biotic and abiotic stresses^16,17,20,22,46–49^. There are three ways of preparing nanoparticles such as chemical, physical and biological known as green chemistry method^50–52^. The physical and chemical methods are time taking, expensive and highly dangerous for releasing toxic chemical while biological method constitute a smart technology with immense potential to revolutionize sustainable agriculture. Their application can lead to improved plant germination, enhanced growth, and increased tolerance to biotic and abiotic stresses, ensuring healthier crops and higher yields^53^. The green synthesized has shown potentials in agricultural production, and its applications include nanoformulations of agrochemicals for applying pesticides and fertilizers for crop improvement^54–56^, the application of nano sensors in crop protection for the identification of diseases and residues of agrochemicals, nanodevices for accurate detection of diseases and nanoliposomes for targeted delivery of gene, drug and peptides^57–62^. Boosting agriculture is urgent need of every country as their food demands are increasing due to rapid enhancements in the population. Here, Wheat (morpho-anatomical and biochemical) characterization was observed for simulating phyto-synthesized ZnO Nanoparticles. The overall preparatory plan and their actions in experimental pot experiment have been shown in schematic illustration of Fig. 1**.**

### Characterization of ZnO NPs

**Figure 2:**
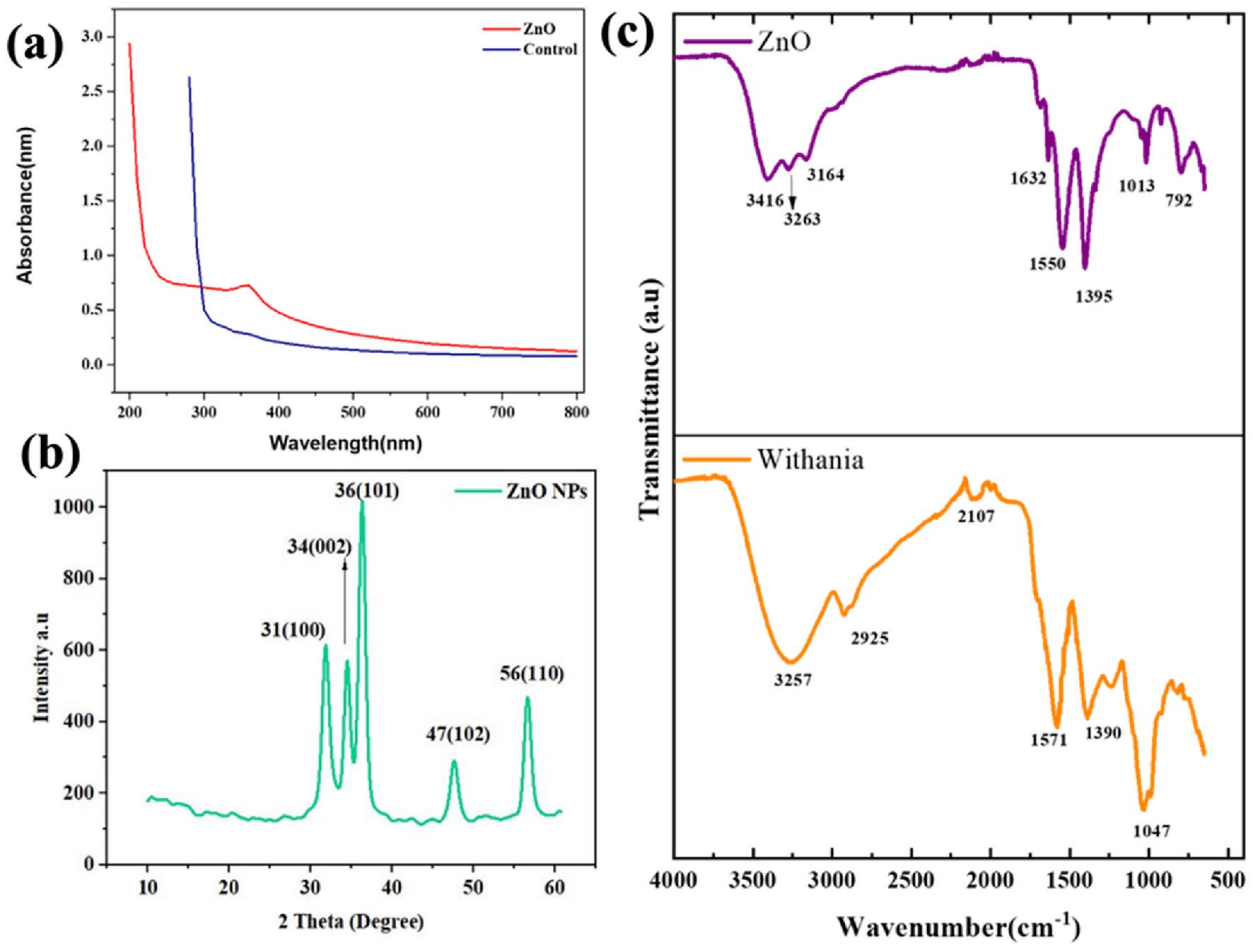
UV–Vis spectrum, XRD and FTIR of ZnO nanoparticles synthesized using *Withania coagulans* extract and FTIR of *Withania coagulans* extract.

UV–Visible absorption spectrum, XRD and FTIR spectrum of *W. coagulans* extract and biologically synthesized ZnO nanoparticles is shown in Fig. 2. The confirmation of stable ZnO Nanoparticles was done by using Uv–Vis spectra of sample solution in the wavelength range of 200-800 nm. The solution containing ZnO NPs showed prominent peaks within the wavelength range of 200 nm to 800 nm for specific element absorbance^63–65^. The distinct peak centered around 350 nm is specific for ZnO NPs Fig. 2a which is due to their large excitation binding energy at room temperature. The XRD pattern of produced ZnO NPs clearly shows crystalline structure for the nanoparticles. Sharp diffraction peaks were found at 2θ values of 31.46, 34.29, 36.33, 47.51, and 56.50 degrees. To identify the numerous distinctive functional groups linked to the developed nanoparticles Fig. 2b. The previously reported data confirmed the current results evidences^66–68^. FT-IR research was performed on zinc nanoparticles. The peaks indicate the functional groups that are typical of the produced zinc oxide nanoparticles. The samples' absorption peaks were determined to lie between 3416 cm^−1^, 3263 cm^−1^, 3164 cm^−1^, 1632 cm^−1^, 1550 cm^−1^, 1395 cm^−1^, 1013 cm^−1^ and 792 cm^−1^. The absorption peak at 792 cm^−1^ corresponds to metal–oxygen (ZnO stretching vibrations) vibration mode Fig. 2c.

### Morpho-anatomical parameters

To study the internal structure of plant cell and their response is very important, here we have observed a lot of changes in cell structure due to stress of nanoparticles which give enough evidence that it is because of our nanoparticle’s simulation^16,69^.

**Figure 3:**
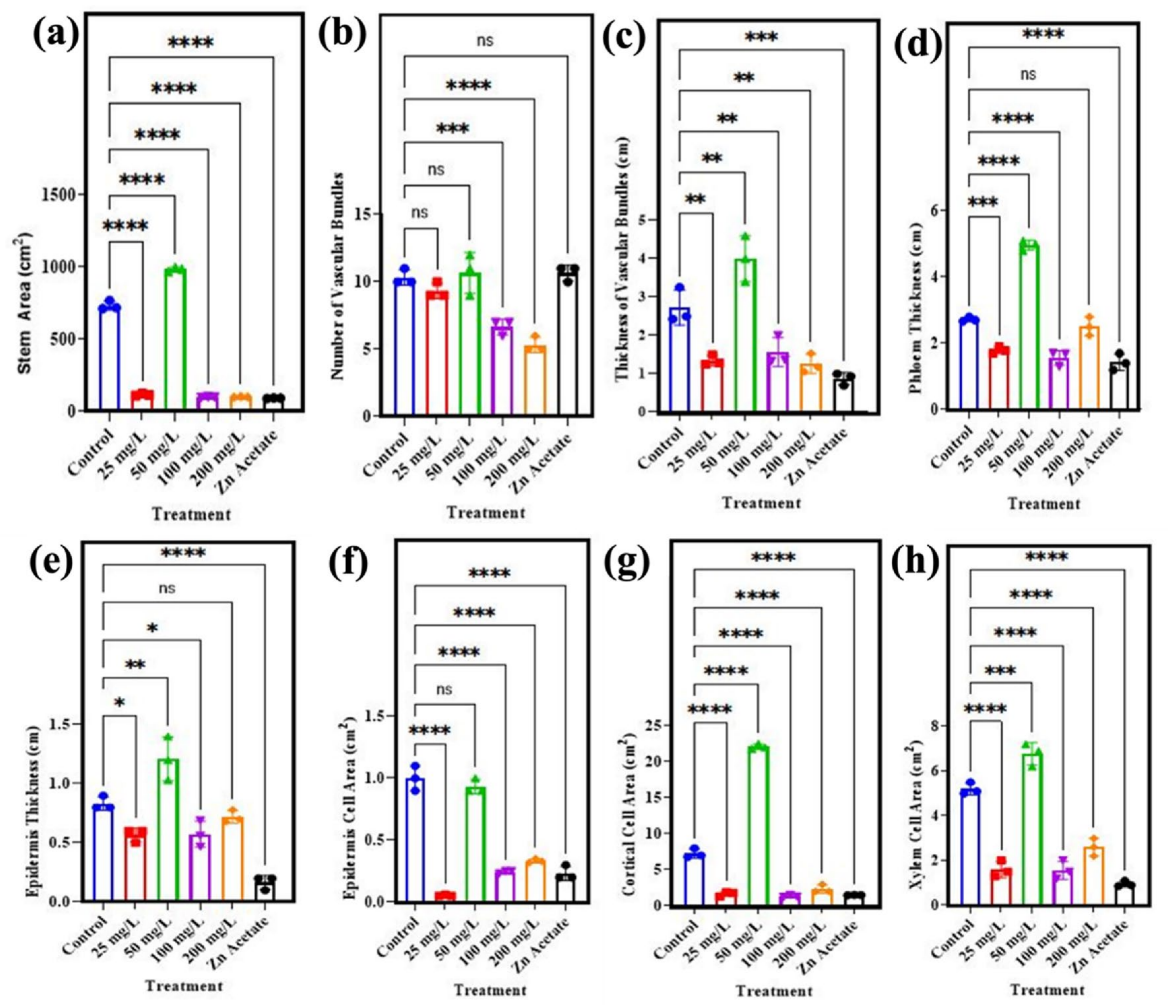
Effect of different concentrations of zinc oxide NPs (0, 25, 50, 100 and 200 mg/l) and Zn acetate on morphological characterization of *Triticum aestivum*. (**a**) Stem area, (**b**) number of vascular bundles, (**c**) thickness of vascular bundles, (**d**) phloem thickness, (**e**) epidermis thickness, (**f**) epidermis cell area, (**g**) cortical cell area, (**h**) xylem cell area.

Anatomical attributes of wheat stem are presented in Fig. 3a–h. The stem area of wheat plant showed significant (p < 0.05) increase of 34% by application of 50 mg/l ZnO NPs as compared to control while other concentration has not shown any significant effect Fig. 3a. The treatment of 50 mg/l ZnO NPs also increased the number of vascular bundles in wheat plants by 3% while the other concentration has shown negative influenced as higher concentration Fig. 3b. In case of thickness of vascular bundles, it has observed that it is significantly (p < 0.05), it has observed the enhanced by 46% Fig. 3c, thickness of the epidermis by 45% Fig. 3e, epidermis cell area by 6% Fig. 3f, cortical cell area by 204% Fig. 3g, thickness of phloem by 82% Fig. 3d and xylem cell area by 30% Fig. 3h.

**Figure 4:**
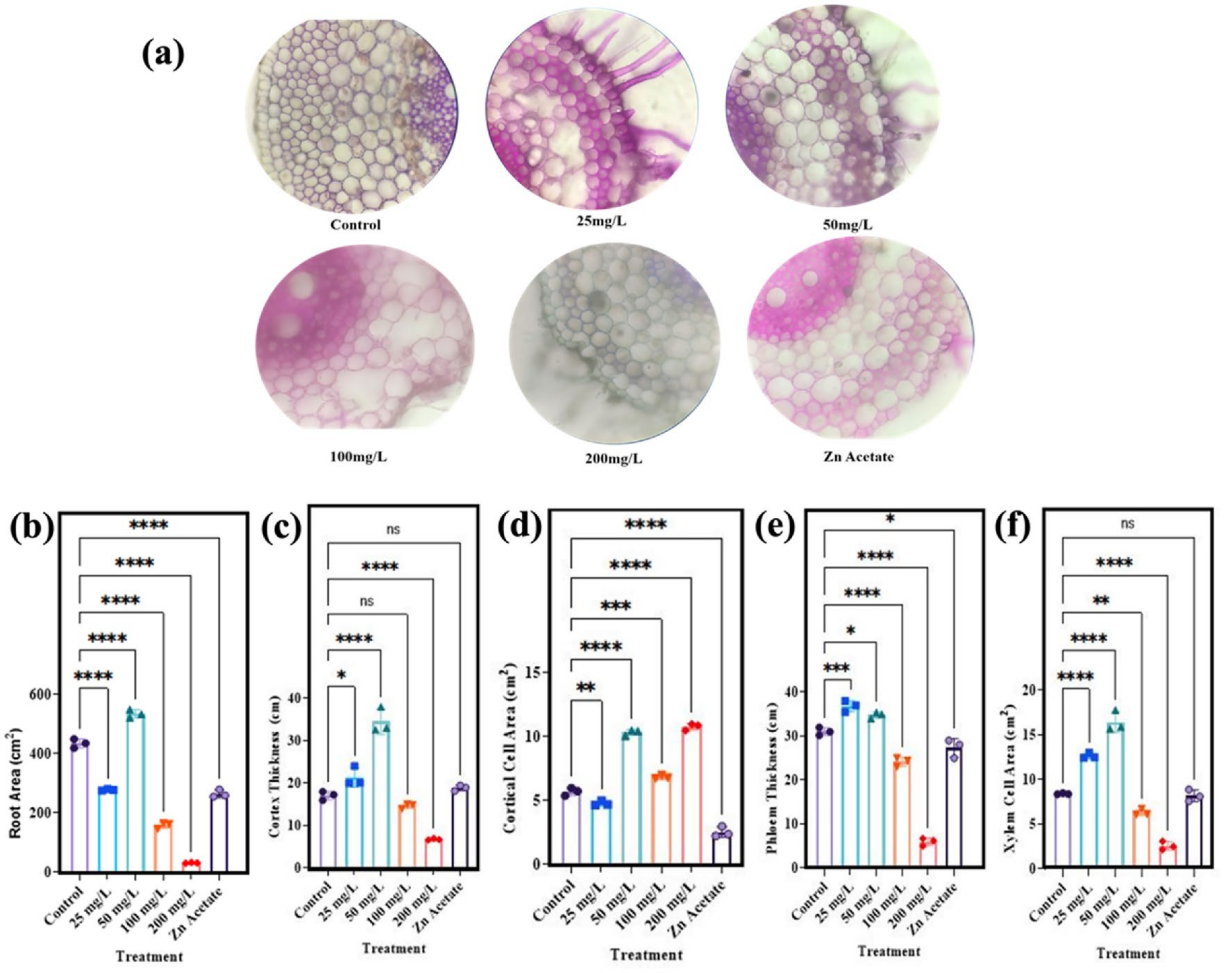
Effect of different concentrations of zinc oxide nanoparticles (0, 25, 50, 100 and 200 mg/l) and Zn acetate on root anatomy of *Triticum aestivum*, (**a**) transvers section of root, (**b**) root area, (**c**) cortex thickness, (**d**) cortical cell area, (**e**) phloem thickness, (**f**) xylem cell area.

**Figure 5:**
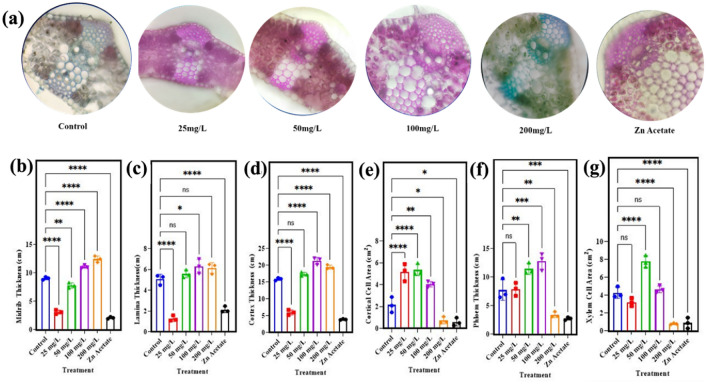
Effect of different concentrations of zinc oxide nanoparticles (0, 25, 50, 100 and 200 mg/l) and Zn acetate on leaf anatomy of *Triticum aestivum*. (**a**) Midrib thickness of leaf, (**b**) leaf thickness, (**c**) cortex thickness, (**d**) cortical cell area, (**e**) phloem thickness, (**f**) xylem cell area (**g**).

The current research was matched with previously reported data which showed the validity of the work and also effectiveness of nanoparticles as fertilizers and their application^70^. Anatomical attributes of wheat root are critical point due to absorbance water and salt which maintain the plant regulation and functionality. To main it’s all activities, we have observed the changes take places with in the root due to influence of nanoparticles induction. Figure 4 presented the root area, thickness xylem and phloem attributes. Changes in the morphology of root due to influence of nanoparticles has shown in Fig. 4a while the root area of wheat plant showed significant (p < 0.05) increase of 23% by application of 50 mg/l ZnO NPs as compared to control plant while other concentration has negligible effect Fig. 4b. The thickness of cortex in root of wheat plant also showed significant (p < 0.05) increase of 101% Fig. 4c, cortical cell area showed significant (p < 0.05) increase of 81% Fig. 4d, thickness of phloem showed increase of 12% Fig. 4e and xylem cell area in root of wheat plant showed increase of 95% Fig. 4f by application of 50 mg/l ZnO NPs. Similarly, the other very important organ is leaf which is considered to be the food factory and its efficiency is main survival point of the plant therefore, leaf study is more important than others organ. For anatomical attributes and the cell morphology of wheat leaves are presented in Fig. 5a. The findings revealed that application of ZnO nanoparticles significant (p < 0.05) increase thickness of midrib in leaf of wheat plant by 22% when exposed to 100 mg/l and 37% when 200 mg/l was applied Fig. 5b. The thickness of lamina in leaf of wheat plant showed increase of 10% by application of 50 mg/l ZnO NPs Fig. 5c. The findings revealed that application of ZnO nanoparticles increase thickness of lamina in leaf of wheat plant by 24% when exposed to 100 mg/l and 21% when 200 mg/l was applied Fig. 5c. The thickness of cortex in leaf of wheat plant showed significant (p < 0.05) increase of 9% Fig. 5d, cortical cell area showed increase of 148% Fig. 5e, thickness of phloem showed increase of 47% Fig. 5f by application of 50 mg/l ZnO NPs. The findings revealed that application of ZnO nanoparticles increase thickness of phloem in leaf of wheat plant by 64% Fig. 5f when exposed to 100 mg/l and decrease of 55% when 200 mg/l was applied. The xylem cell area in leaf of wheat plant showed increase of 81% Fig. 5g by application of 50 mg/l ZnO NPs.

### Growth and yield parameters

**Figure 6:**
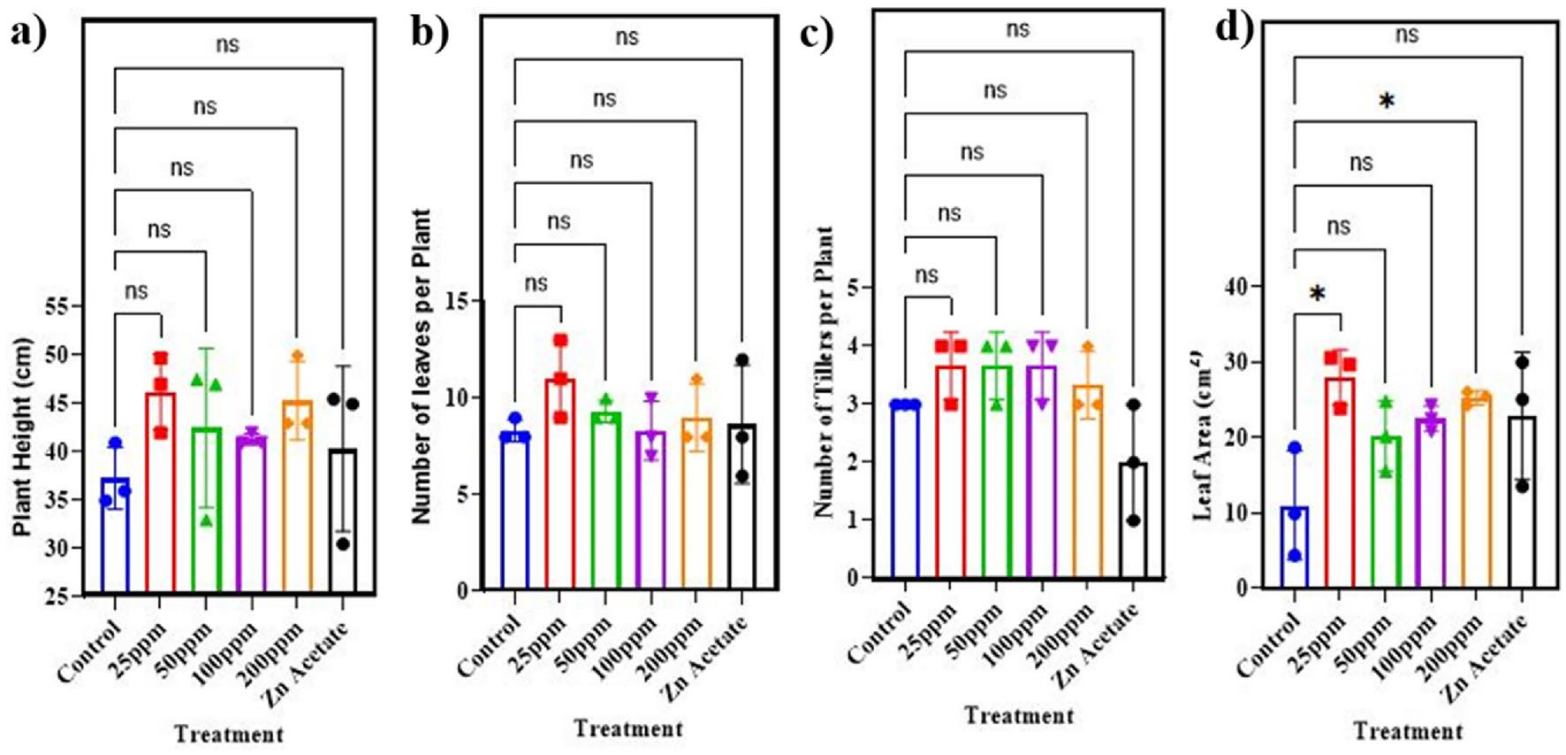
Effect of different concentrations of zinc oxide nanoparticles (0, 25, 50, 100 and 200 mg/l) and Zn acetate on (**a**) plant height, (**b**) number of leaves per plant, (**c**) number of tillers, (**d**) leaf area of *Triticum aestivum*.

**Figure 7:**
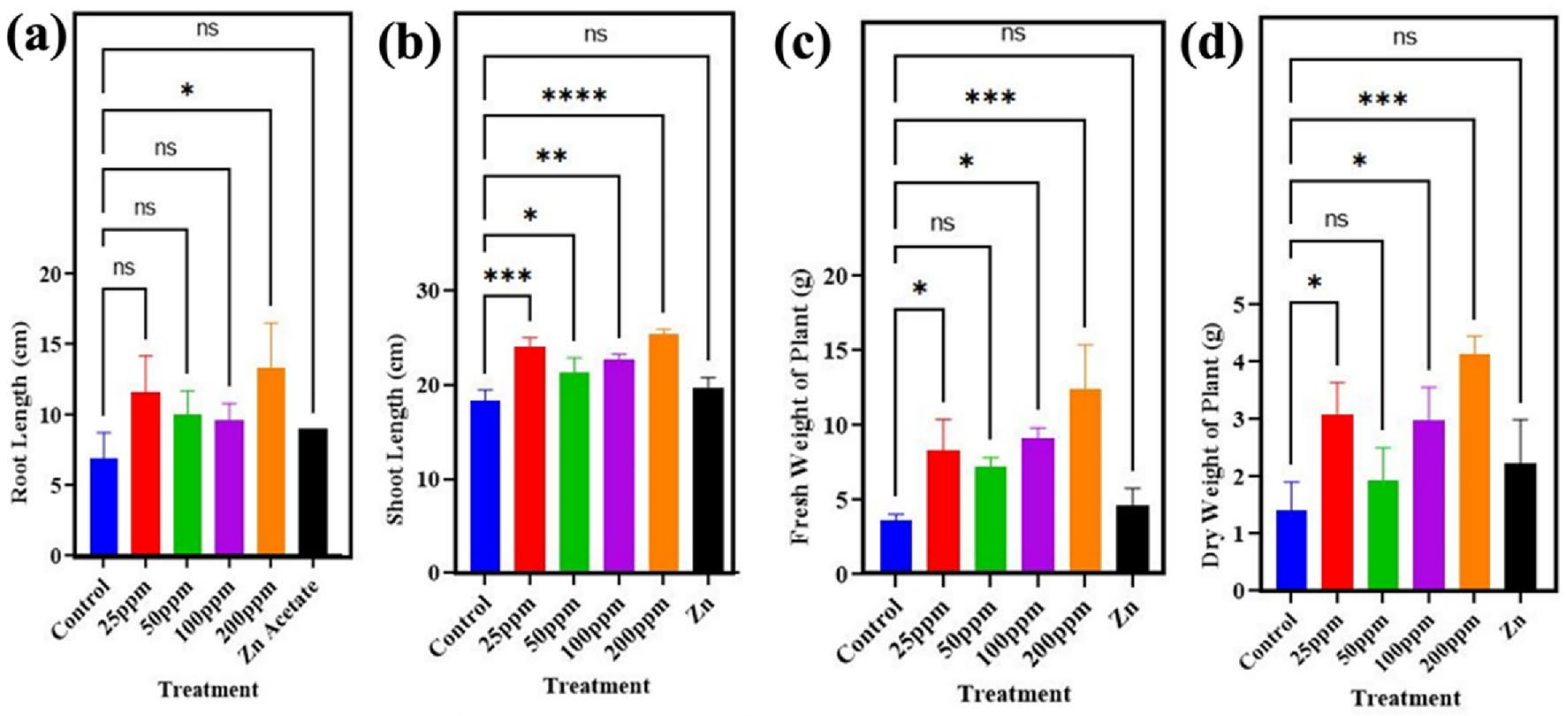
Effect of different concentrations of zinc oxide nanoparticles (0, 25, 50, 100 and 200 mg/l) and Zn acetate on (**a**) root length, (**b**) shoot length, (**c**) fresh weight, (**d**) dry weight of *Triticum aestivum.*

**Figure 8:**
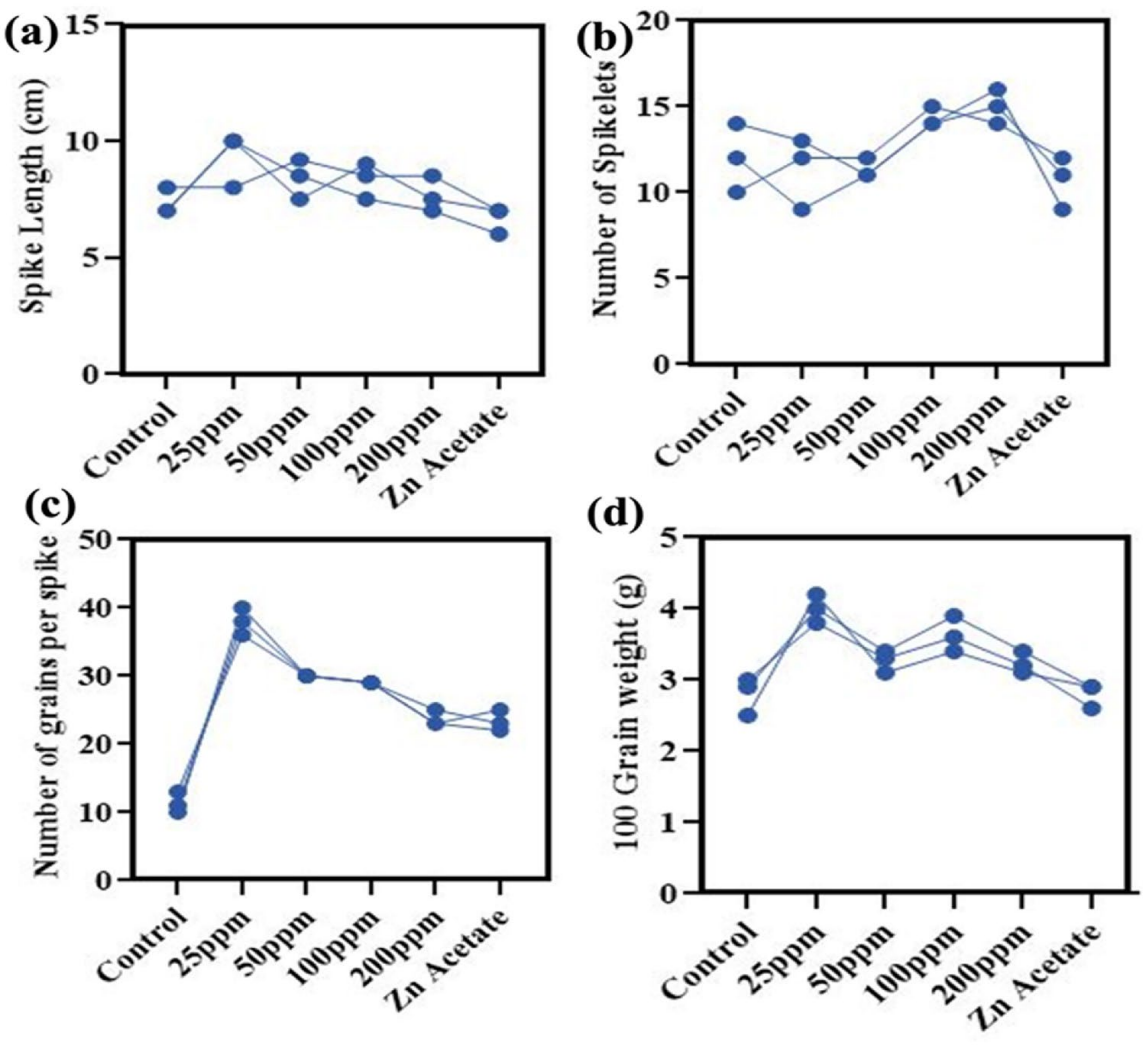
Effect of different concentrations of zinc oxide nanoparticles (0, 25, 50, 100 and 200 mg/l) and Zn acetate on (**a**) spike length, (**b**) number of spikelet’s per spike, (**c**) number of grains per spike, (**d**) 100 grain weight of *Triticum aestivum.*

The plant growth attributes of wheat were recorded to explore the effects of ZnO NPs on wheat growth as previously reported^71,72^. The results pertaining to plant height, number of leaves, number of tillers, leaf areas, root length, shoot length, fresh weight, dry weight, spike length, number of spikelet’s, number of grains per spike and 100 grain weight are depicted in Figs. 6, 7 and 8. In case of plant height, the data was obtained and observed that the greatest plant height was attained by using 25 mg/l of ZnO NPs. The greatest significant (p<0.05) increase in plant height was 23% when compared to the control. While 50 mg/l, 100 mg/l, and 200 mg/l applications resulted in substantial increases of 13.8%, 10.7%, and 21.4%, respectively Fig. 6a.

The obtained data indicated that number of leaves per plant increased significantly when wheat plant was exposed to different concentrations of ZnO NPs. At 25 mg/l concentration, number of leaves increased to 32%. The treatment of 50 mg/l and 200 mg/l also increased the number of leaves in wheat plant by 12% and 8% significantly Fig. 6b. The treatment of plant with 25 mg/l showed increase of 153% in leaf area as compared to control. 104% and 129% increase in leaf area was noticed at 100 mg/l and 200 mg/l ZnO nanoparticles. 83% increase was observed when wheat plant was exposed to 50 mg/l of ZnO nanoparticles Fig. 6d. This study found that ZnO NPs had a stronger influence on plant development and physiology than Zn acetate salt. Nanoparticles are more efficiently absorbed and processed by seeds due to their smaller size and reduced solubility. Biomass generation is a good indication of plant photosynthetic processes. The study analyzed biomass production based on root and shoot length and fresh and dry weights.

Furthermore, the addition of ZnO NPs resulted in dramatic significant (p < 0.05) increase in root growth, also, 93% increase in root length was observed by application of 200 mg/l ZnO NPs. 25 mg/l of ZnO NPs showed 69% and 100 mg/l showed 40% increase of root length in seedling as compared to control Fig. 7a. While increase of 44.9% was noticed at concentration level of 50 mg/l of ZnO NPs. 200 mg/l alone showed increase of 38.9% in shoot length as compared to control Fig. 7b. While 30%, 16.3% and 23.6% increase was observed at 25 mg/l, 50 mg/l and 100 mg/l, respectively in comparison to control. A remarkable increase of 241% and 18% of fresh weight was noticed when plants were treated with 200 mg/l and 25 mg/l. While 151% increase was observed at 100 mg/l as compared to control Fig. 7c. The dry weight of wheat plant showed significant (p<0.05) increase of 120% by application of 25 mg/l ZnO NPs. While 50 mg/l and 100 mg/l showed increase of 38% and 112% in comparison to control. While the highest value of increase of 194.3%o was noticed at application of 200 mg/l ZnO nanoparticles Fig. 7d. The result presented by Imtiaz (2003) aligns with our findings that zinc increases fresh weight and dry weight of wheat seedlings.

The application of 25 mg/l of ZnO NPs showed 27.2% significant (p < 0.05) increase in spike length of wheat plant. On the other side, relative to control 50 mg/l, 100 mg/l and 200 mg/l also showed positive impacts on spike length *i.e.* 14%. 13% and 4.5%, respectively Fig. 8a. Data presented showed the positive impact of higher doses of ZnO nanoparticles on number of spikelet’s per spike in wheat variety. Alone 200 mg/l of ZnO NPs showed increase of 25%. While 100 mg/l of ZnO NPs also upregulated this character in wheat by 19.4%. While application of zin acetate stress, this feature reduces by 11% that shows the toxic effects on yield attributes Fig. 8b. Exogenously applied ZnO NPs on wheat plants observed a remarkable increase in number of grains per spike Fig. 8c. Results illustrated that the trace level of ZnO NPs 25 mg/l and 50 mg/l promotes plant growth and grain yield by 235% and 164%, respectively. While significant (p < 0.05) increase of 155% and 108% was observed on application of 100 mg/l and 200 mg/l of ZnO nanoparticles. However, the grain weight of plant at 25 mg/l showed an evident increase of 42% after the ZnO NPs application, as compared to control Fig. 8d. The most significant increase in grain weight was documented in wheat plants provided by 50 mg/l and 100 mg/l of ZnO NPs, i.e. 16.6% and 29.7%. While alone 200 mg/l of ZnO NPs showed 15.4% increase in weight of grains obtained from wheat plants. The increase in biomass could be due to Zn ions in ZnO NPs treated plants have an important function in the manufacture of natural auxin (IAA) in plants, leading to increased cell division and enlargement. This might explain the rise in biomass.

### Photosynthetic pigments

**Figure 9:**
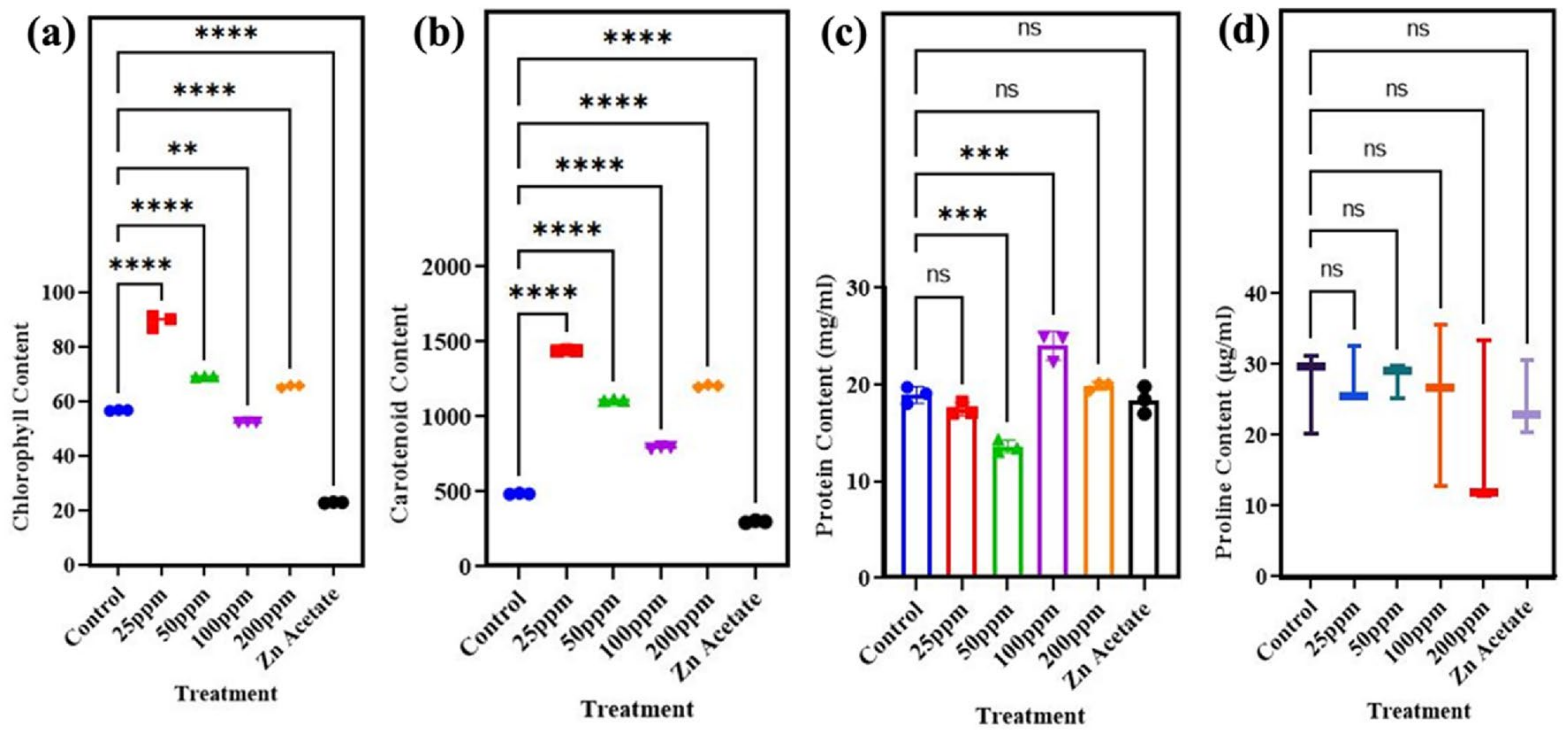
Effect of different concentrations of zinc oxide nanoparticles (0, 25, 50, 100 and 200 mg/l) and Zn acetate on (**a**) chlorophyll content, (**b**) carotenoid content, (**c**) protein content, (**d**) proline content of *Triticum aestivum.*

Chlorophyll concentration correlates positively with photosynthetic rate. Thus, a change in total level of chlorophyll may be utilized to assess plant health. Data of chlorophyll and carotenoid content in fresh leaves of wheat plant depicted in Fig. 9. Response of wheat plant in terms of total chlorophyll content was significantly upregulated in zinc oxide nanoparticle treated plants. The significant (p < 0.05) increased phytochemical content was observed at 25 mg/l and 50 mg/l i.e. 57% and 21.8% respectively, as that of control Fig. 9a. Leaf chlorophyll content was significantly improved by 15% when exposed to 200 mg/l ZnO NPs. When exposed to 25 mg/l and 200 mg/l under regulated irrigation circumstances, the maximum carotenoid levels were recorded as 196% and 147%, respectively Fig. 9b. When 50 mg/l and 100 mg/l ZnO NPs were foliar sprayed at different stages of the wheat plant, the carotenoid content increased by 128% and 62%, respectively. The recorded increase in chlorophyll content in ZnO NPs treated plants can be attributed to the increase in water uptake and nutrient absorption in presence of Zn, resulting in better seedling growth and physiological performance as compared to control. ZnO NPs may have a favorable impact on chlorophyll content because Zn protects the sulfhydryl group of chlorophyll molecules during production. Current study found that ZnO NPs boosted chlorophyll content in NP plants. Increasing the chlorophyll content, a key factor in plant photosynthetic efficiency, led to an overall rise in biomass in ZnO NP plants.

Zinc ion in ZnO Nps helps to maintain bio-membranes by accumulating phospholipids and promotes protein synthesis in plant cells. Data related to protein and proline content in fresh leaves is presented in Fig. 9c, d. The findings revealed that application of ZnO nanoparticles enhanced (p < 0.05) level of protein in wheat plant by 26.8% when exposed to 100 mg/l and 3.9% when 200 mg/l was applied. While a great decline of 7% and 28% was observed when plant was foliar sprayed with low doses of nanoparticles i.e. 25 mg/l and 50 mg/l of ZnO NPs Fig. 9c. Zinc acetate100mg/l dose application also cause reduced level of protein by 2%. Proline content of wheat plants was significantly affected by application of ZnO NPs. Proline content was upregulated by 2.79% and 3.5% when 25 mg/l and 50 mg/l of ZnO NPs were applied Fig. 9d.

### Antioxidant enzyme activities

**Figure 10:**
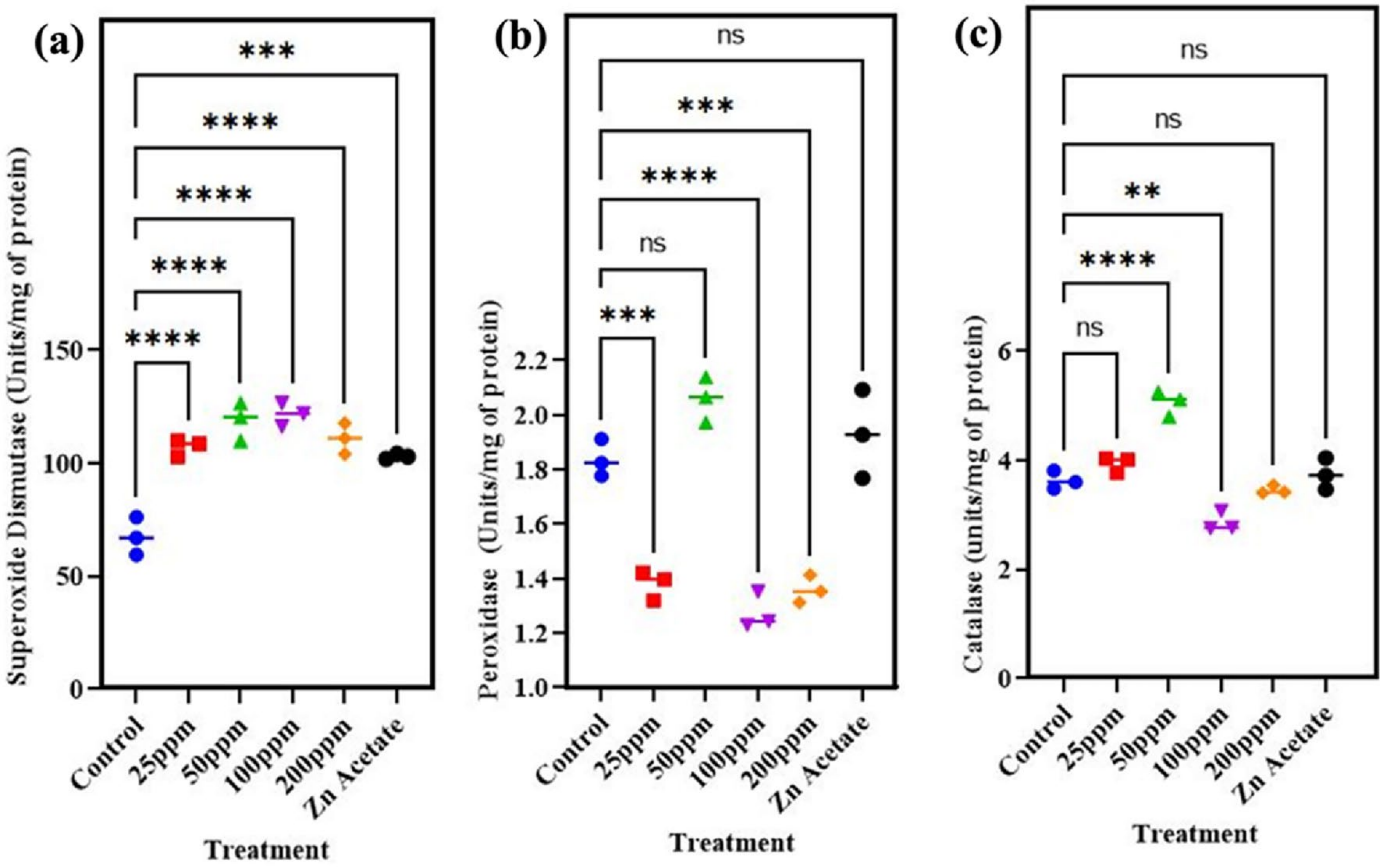
Effect of different concentrations of zinc oxide nanoparticles (0, 25, 50, 100 and 200 mg/l) and Zn acetate on (**a**) superoxide dismutase (**b**) peroxidase, (**c**) catalase of *Triticum aestivum*.

The defense system of any organism is very important due to survival of living organism. To cope with all kinds of oxidative stresses or growth promoters trigger the defense system. For this purpose, the data related to SOD, POD and CAT activities in leaves has been reported in Fig. 10a–c. Peroxidase (POD) scavenges reactive oxygen species that cause cell oxidative damage. Plant peroxidases serve as biochemical indicators for various abiotic stressors. The peroxidase (POD) level of wheat plant showed increase of 12% by application of 50 mg/l ZnO NPs Fig. 10a. The wheat plants under the treatment of ZnO nanoparticles showed significant (p<0.05) increase in superoxide dismutase (SOD) Fig. 10b. Relative to control, the maximum increase in SOD was recorded 79.7% at 100 mg/l of ZnO NPs. Increment in SOD was 58% and 75% at 25 mg/l and 50 mg/l was also recorded. While 200 mg/l of ZnO NPs alone showed increase of 64% in SOD level in leaves of wheat plant Fig. 10c.

CAT protects against oxidative damage to cells. CAT can scavenge bulk H_2_O_2_, whereas peroxidases can sequester any residual H_2_O_2_. SOD converts O_2_ to H_2_O_2_, while CAT decomposes H_2_O_2_ into water and oxygen molecules. The results show that application of ZnO nanoparticles enhanced level of catalase (CAT) in wheat plant by 39% when exposed to 50 mg/l and 8.4% when 25 mg/l was applied. Our pot experiment results were in synchronization with ^73^ whose findings suggested that foliar exposure of ZnO NPs at different time periods during growth has significant impact on plant growth. Treatment with ZnO NPs boosted up the level of photosynthetic pigments and oxidative stress of plant and increased the leaf POX and SOD activities than control^4^. As it is well known that main role of chlorophyll is to absorb light and convert it into chemical and electronic energy. Suitable increase in chlorophyll content is favorable by plants^74,75^. Our results suggested that at 25 and 50 mg/l concentration of ZnO nanoparticles, chlorophyll increased by 57% and 22% over control. However, at 100 mg/l of ZnO nanoparticles shown significant decrease in total chlorophyll content. Significant impact of ZnO nanoparticles has been reported by ^76^ when plants were exposed to ZnO nanoparticles at dose of 300 mg/kg, plant showed maximum increase in dry weight of root, shoot and grain^77^. Nanoparticle application led to increase in proline content of plant. Our studies showed 3% and 4% increase at 25 and 50 mg/l of ZnO NPs that is in accordance with ^78^ whose studies suggested that proline content in leaves increased 3.3 and 4.5-fold in wheat while 1.7 and 2.2-fold increased in tomato when subjected to 200 mg Zn/l.

**Figure 11:**
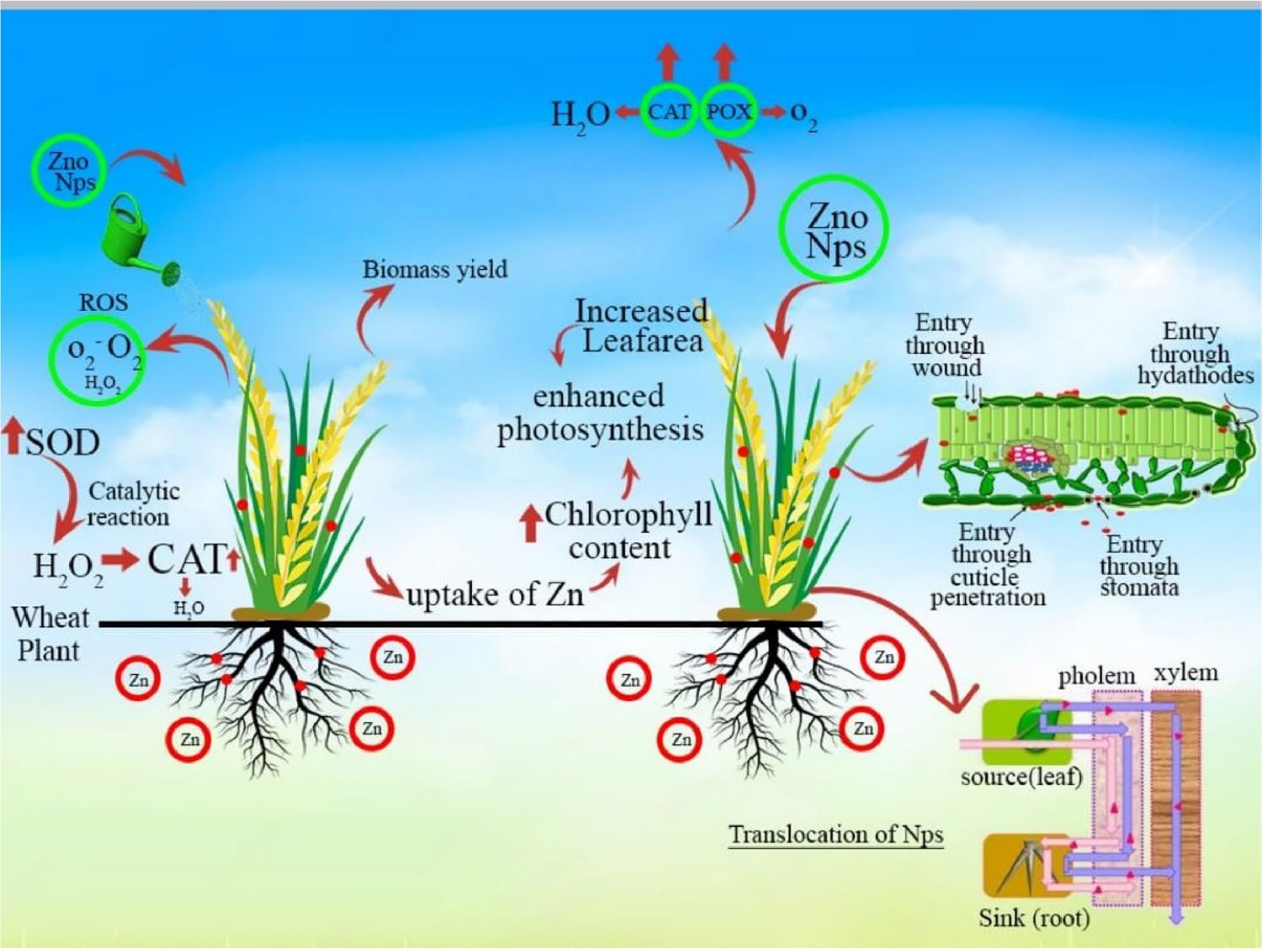
Purpose mechanisms underlying the observed effects of ZnO NPs on wheat growth.

The use of Zinc Oxide Nanoparticles (ZnO NPs) in agriculture holds promise for various applications such as crop protection, nutrient delivery, and soil remediation ^64,79^. However, there are also potential risks and limitations associated with their use. One of the primary concerns is the potential for ZnO NPs to accumulate in the environment. If not properly managed, they could leach into soil and water systems, leading to unintended consequences such as soil contamination or adverse effects on aquatic organisms. While zinc is an essential micronutrient for plants, excessive levels can be toxic. Figure 11 showed the possible potential mechanism underlying the observed effects of ZnO nanoparticles on wheat growth There is a risk that the use of ZnO NPs could lead to zinc accumulation in plants, disrupting their physiology and potentially affecting human health if consumed. Moreover, there may be unintended consequences for non-target organisms in the environment. There are concerns about the potential health risks associated with exposure to ZnO NPs for agricultural workers and consumers. Inhalation of nanoparticles during application or ingestion of contaminated products could pose health hazards, although the extent of these risks is still being studied. There is a possibility that prolonged use of ZnO NPs could lead to the development of resistance in pests, reducing the effectiveness of nanoparticle-based pesticides over time. Additionally, their ecotoxicological effects on soil microorganisms, beneficial insects, and other components of the ecosystem need to be thoroughly assessed.

## Conclusions

The primary goal of this study was to look into the effects of phyto- generated ZnO nanoparticles on growth and development of wheat plant. Effective results depend on concentration of nanoparticles used. In wheat seeds, ZnO NPs at a concentration of 25 mg/l enhanced stem, root, and leaf anatomy by far any concentration tested. ZnO nanoparticles spray at 25 mg/l concentration improved morphological, physiological, biochemical, and enzymatic activity during wheat crop growth. ZnO nanoparticles were shown to be more efficient in increasing wheat plant growth, yield, and Zn biofortification. The total performance index in ZnO NPs treated plants was found to be significantly enhanced. This helped the ZnO NPs treated plants to improve the overall primary photochemistry which is directly correlated with improved biomass accumulation. They may also be effective in improving crop nutritional quality. This fertilizer would be needed in modest amounts, reducing environmental contamination and promoting environmentally friendly agriculture. New findings may lead to more efficient agricultural practices and result in higher yields with fewer inputs, reducing the environmental footprint of farming operations. Understanding the findings can help farmers and policymakers make decisions that minimize negative environmental impacts, such as reducing greenhouse gas emissions, preventing soil erosion, and preserving biodiversity. The findings can inform strategies for making agriculture more sustainable in the long term. Sustainable agricultural practices can also have economic benefits for farmers, such as lower production costs, access to premium markets for organic or sustainably produced goods, and improved resilience to market fluctuations.

## Data Availability

All the data generated/ analyzed during the study are available with the corresponding author on reasonable request.
